# Malnutrition and Opportunistic Infections among People Living with HIV Receiving Anti-retroviral Therapy in Aceh, Indonesia

**Published:** 2018-05

**Authors:** Cut LLF PUTRI, Masra L. SIREGAR, Harapan HARAPAN, Husnah HUSNAH

**Affiliations:** 1. Medical Research Unit, School of Medicine, Syiah Kuala University, Banda Aceh, Indonesia; 2. Dept. of Internal Medicine, School of Medicine, Syiah Kuala University, Banda Aceh, Indonesia; 3. Dept. of Internal Medicine, Dr. Zainoel Abidin Hospital, Syiah Kuala University, Banda Aceh, Indonesia; 4. Tropical Disease Centre, School of Medicine, Syiah Kuala University, Banda Aceh, Indonesia; 5. Dept. of Microbiology, School of Medicine, Syiah Kuala University, Banda Aceh, Indonesia; 6. Dept. of Nutrition, School of Medicine, Syiah Kuala University, Banda Aceh, Indonesia

## Dear Editor-in-Chief

In 2015, the number of people living with HIV (PLHIV) receiving antiretroviral therapy (ART) reaching 17.0 million people (1). Globally, scale-up of ART has surpassed expectations, but ART is not without adverse effects. It causes nausea, vomiting, loss of appetite, diarrhea, and therefore affects nutritional status. The prevalence of malnutrition among receiving ART was substantially high (2). In addition, the incidence of HIV-associated opportunistic infections (OIs) is still a major problem in the era of highly active ART (3). Although the prevalence of PLHIV in Aceh is significantly increased (4) and social aspects of PLHIV have been reported (5, 6), study regarding clinical and nutritional aspects of PLHIV from this area is unavailable. Therefore, this study was conducted to assess the prevalence of malnutrition and type of OI among PLHIV in Aceh, Indonesia.

This cross-sectional study was conducted at Voluntary, Counseling, and Testing (VCT) clinic of Dr. Zainoel Abidin Hospital, Banda Aceh, Indonesia from 1 Jul to 30 Nov 2016.

Nutritional information was collected using Mini Nutritional Assessment-directed interview and anthropometric measurements of body composition. Basic demographics and clinical data, and information about ART (the length and regimens) and data on OI were retrieved from patient records. The association of ART duration and nutritional status was assessed using Spearman correlation.

This study was approved by the Ethics Committee of School of Medicine, Syiah Kuala University, Banda Aceh, Indonesia.

Forty PLHIVs were included and analyzed. The characteristics of the participants are presented in Table 1.

**Table 1:** Characteristics, type opportunistic infection and prevalence of malnutrition among the people living with HIV in Aceh (*n*=40)

***Characteristic***	***Frequency (%)***
Gender	
Male	25 (62.5)
Female	15 (37.5)
Age(yr)	
18–25	6 (15.0)
26–35	22 (55.)
36–45	9 (22.5)
46–55	3 (7.5)
Duration of HIV infection	
< 1 tahun	13 (32.5)
1–2 tahun	13 (32.5)
3–4 tahun	9 (22.5)
≥5 tahun	5 (12.5)
HIV transmission route	
Heterosexual	26 (65.0)
Homosexual	11 (27.5)
Bisexual	1 (2.5)
Healthcare-related	1 (2.5)
Injection drug	1 (2.5)
Antiretroviral therapy regimens	
AZT + 3 TC + NVP	27 (67.5)
AZT + 3 TC + EFV	4 (10)
TDF + 3 TC + NVP	7 (17.5)
TDF + 3 TC + EFV	2 (5.0)
Duration of ART	
Less than 3 months	9 (22.5)
≥3 months	31 (77.5)
Opportunistic infection(s)	
Pulmonary tuberculosis	7 (17.5)
Extrapulmonary tuberculosis	4 (10.0)
Chronic diarrhea	9 (22.5)
Oral candidiasis	4 (10.0)
Seborrheic dermatitis	1 (2.5)
Pulmonary tuberculosis + oral candidiasis	3 (7.5)
Seborrheic Dermatitis + oral candidiasis	1 (2.5)
Chronic diarrhea + oral candidiasis	5 (12.5)
Chronic diarrhea + Toxoplasmosis	1 (2.5)
Pulmonary tuberculosis + chronic diarrhea + oral candidiasis	3 (7.5)
Chronic diarrhea + oral candidiasis + meningitis	1 (2.5)
Oral candidiasis + herpes + seborrheic dermatitis	1 (2.5)
Nutritional status^*^	
Normal nutritional status	10 (25.5)
At risk of malnutrition	23 (57.5)
Malnourished	7 (17.5)

AZT: Zidovudine, EFV: Efavirenz, NVP: Nevirapine, 3TC: Lamivudine or epivir, TDF: Tenofovir disoproxil fumarate

* Based on Mini Nutritional Assessment (MNA)

Majority of the participants (62.5%) was male and most of them were infected via heterosexual transmission (65.0%). All of the participants had current or past history of OI, of which chronic diarrhea was the most predominant (22.5%) followed by pulmonary tuberculosis (17.5). Only approximately a quarter of the participants were had normal nutritional status while 17.7% and 57.5 of them were malnourished and at risk of malnutrition, respectively. Spearman correlation test revealed a substantial correlation between ART duration and malnutrition (r=0.397, *P*=0.011).

This study suggests that there is correlation between the lengths of ART and the prevalence of malnutrition among PLHIV receiving ART in Aceh.

Therefore, regular screening and evaluation of nutritional status are important. Education and training to improve dietary are important to be conducted because adequate and diversified nutrition is necessary to manage OIs, maintain the immune system, optimize response to medical treatment, and support optimal quality of life in PLHIV (7). A standardized nutritional supplement along with ART could be implemented. Nutritional supplementation with ART was improved not only immune response and weight gain but also drug adherence and physical activity among PLHIV in South Africa (8). Besides, strengthening household food security status of PLHIV and regular checkup of simple indication of OIs among PLHIV in general and those taking ART, in particular, is recommended.
